# Gynaecological Laparoscopic Surgeries in the Omicron Era: Improvising the Management Skills After Lessons Learnt From the Delta Wave

**DOI:** 10.7759/cureus.29904

**Published:** 2022-10-04

**Authors:** Avir Sarkar, K K Roy, Rinchen Zangmo, Maninder K Ghotra, Anshul Kulshreshtha, Ashmita Saha, Nilanchali Singh, Deepika Kashyap

**Affiliations:** 1 Obstetrics and Gynaecology, Employees’ State Insurance Corporation (ESIC) Medical College and Hospital, Faridabad, Faridabad, IND; 2 Obstetrics and Gynaecology, All India Institute of Medical Sciences, New Delhi, New Delhi, IND

**Keywords:** laparoscopy, sars-cov-2, minimally invasive gynaecological surgeries, omicron wave, covid-19 retro

## Abstract

Background

During the peak of the Omicron wave, elective laparoscopic surgeries were restricted; however, semi-emergency and emergency cases were managed despite the limited resources and manpower. We conducted this study to assess the types of gynaecological laparoscopic surgeries performed, the difficulties faced during the Omicron wave, and how we could implement the lessons learnt from the previous Delta wave for better management of gynaecological cases in the Omicron wave.

Methodology

We conducted a prospective cohort study over a period of three months involving 105 patients who underwent laparoscopic surgeries. Based on the decision regarding surgical incision time, the surgeries were sub-classified into immediate, urgent, and expedited. The surgical outcome and satisfaction rates among the patients were assessed through various parameters.

Results

Most of the women (81.9%) were pre-menopausal. Diabetes and chronic hypertension were the predominant medical co-morbidities. Three patients had a history of cardiac valve replacement which required switching warfarin to unfractionated heparin in the pre-operative period. Nearly three-fourthsof the study patients were doubly vaccinated against coronavirus disease 2019 (COVID-19) (77; 73.4%). A total of 14 (13.3%) patients had a history of COVID-19 infection in the past two weeks prior to the current admission. Immediate, urgent, and expedited surgeries comprised 11.4%, 22.8%, and 65.8% of total surgeries, respectively. On assessing the ease of pre-operative preparation according to the five-point Likert scale, immediate, urgent, and expedited surgeries were rated with a mean score of two, four, and five, respectively. The mean duration of surgery in the immediate and urgent groups was 37.6 and 44.2 minutes, respectively. The expedited group comprising mostly laparoscopic myomectomies and hysterectomies required an average duration of 92.6 minutes. The mean rating of patient satisfaction measured by the Likert scale was four, five, and five, respectively, in the three subgroups. Pre-operative patient preparation during the Omicron wave was faster, thereby decreasing the decision to incision interval compared to the Delta wave.

Conclusions

The lessons learnt from the previous Delta wave were used to modify the existing hospital policies in the Omicron wave. More number of vaccinated ground staff, less stringent intubation and extubation protocols during surgery, and lesser duration of post-operative stay helped modify our existing hospital policies for better patient care and satisfaction.

## Introduction

The coronavirus disease 2019 (COVID-19) pandemic is a public crisis. With a surge in cases during each COVID-19 wave, elective surgeries had to be postponed for the diversion of resources toward pandemic control [1,2]. However, emergencies were operating as before. The Omicron wave has been milder than the previous Delta wave [3,4]. Elective laparoscopic surgeries were restricted; however, semi-emergency and emergency cases were managed despite the limited resources and manpower. In this study, we assessed the types of laparoscopic surgeries performed, the difficulties faced during the Omicron wave, and how we could implement the COVID-19-appropriate management skills learnt from the previous Delta wave [5].

## Materials and methods

This prospective cohort study was conducted in the Division of Minimally Invasive Gynecological Surgery at the All India Institute of Medical Sciences over three months during the Omicron wave in India. Institutional Ethics Committee approval was obtained prior to the commencement of the study (IEC-204/2022). Written informed consent was obtained from all participants. Patients undergoing gynaecological laparoscopic surgeries due to emergency and semi-emergency causes were included in the study. Gynaecological malignancies requiring staging laparotomies and debulking surgeries, other open surgeries, and obstetric emergencies other than ectopic pregnancies were excluded. All confirmed COVID-19-positive patients usually deferred surgery or were operated on in COVID-19-dedicated operation theatres depending on the nature of the emergency and were also excluded from the study. Pre-surgical screening protocol was partially modified during the milder Omicron wave. After an initial assessment by the resident doctors, COVID-19 testing was performed from nasopharyngeal swabs through either reverse transcriptase polymerase chain reaction (RT-PCR) or cartridge-based nucleic acid amplification test (CB-NAAT). Patients requiring immediate surgical interventions were operated on after performing a rapid antigen test (RAT), with all COVID-19-appropriate surgical precautions in place. All surgeries were performed by the same laparoscopic surgeon to eliminate bias. Proper personal protective equipment (PPE) was ensured in all surgeries. In each case, disposable trocars were used. Intra-abdominal pressures were maintained as low as feasible (10-12 mmHg). Energy sources such as harmonic and bipolar cautery were used in low power settings of 30-50 W for bipolar coagulation or using newer devices such as a harmonic ace and ligasure with a less smoke-generating system. Surgical fumes were suctioned intermittently using the closed method. Pneumoperitoneum was deflated prior to delivery of specimen. Proper caution was exercised to avoid spillage of any fluid or blood droplets into the external environment. These COVID-19-appropriate measures were similar to that followed during the Delta wave [5-7].

Considering the infectivity rate of the virus and for better patient care, operative guidelines were modified during the omicron wave. The process of admission to general wards was easier compared to that during the Delta wave. Patients could be admitted to wards without waiting for a documented negative RT-PCR report. Those requiring emergency operations could be taken to operation theatres after a negative RAT report. Moreover, RT-PCR report from laboratories outside the hospital of admission was also considered valid. Patients with a prior history of recovery from COVID-19 infection in the recent past were not refrained from laparoscopic surgeries and admission was considered, unlike the previous protocol of postponing admission for four weeks post-COVID-19 infection [8].

During admission, the vaccination status of all patients was enquired. Admission to surgical incision time was noted in all cases. The laparoscopic surgeries performed were divided into immediate, urgent, and expedited based on the time from decision to surgical incision. Immediate surgeries comprised life-saving interventions where the interval from the decision to surgery was in minutes (less than an hour). Urgent surgeries were performed within hours of diagnosis (less than 24 hours), whereas expedited surgeries took two to three days for pre-operative preparation. Immediate surgeries were performed after a negative RAT which was conducted bedside after admission. Urgent surgeries were done after procuring negative CB-NAAT, while expedited surgeries were performed after negative RT-PCR reports with an average reporting time of 90 minutes and six hours, respectively (owing to the huge burden of samples at the laboratories). An assessment of the ease of pre-operative preparation was done after enquiring from the surgical team and was rated using a five-point Likert scale (very difficult, difficult, neither easy nor difficult, easy, very easy). Anaesthesia time was noted in each case. General anaesthesia was not contraindicated in patients with recent recovery from COVID-19, and pre-intubation computed tomography of the chest was not required. The duration of surgery and the total number of persons assisting the primary surgeon were noted. The enhanced recovery after surgery (ERAS) protocol was followed, and patients were discharged early in the post-operative period [9]. The total duration of the hospital stay was noted. Patient satisfaction was also rated according to the Likert scale (very dissatisfied, dissatisfied, neutral, satisfied, very satisfied) at the time of discharge.

## Results

Participants were recruited prospectively over three months beginning from January 2022 to March 2022 (during the peak of the Omicron wave). A total of 105 patients underwent emergency and semi-emergency gynaecological laparoscopic surgeries during this period. The baseline characteristics are shown in Table 1. The majority of the population (81.9%) was in the pre-menopausal age group. Most women belonged to the upper-middle (33.3%) socio-economic class according to the Modified Kuppuswamy classification. In total, 25 patients were detected to have underlying co-morbidities, of which diabetes and chronic hypertension were the predominant disorders. Three patients had a history of cardiac valve replacement which required switching warfarin to unfractionated heparin in the pre-operative period. Two patients had underlying bronchial asthma which was controlled with short-acting beta-agonists. A patient who was diagnosed with steroid-dependent rheumatoid arthritis required a stress dose of hydrocortisone in the peri-operative period. These patients required a longer duration for pre-operative assessment and anaesthetic preparation. Nearly three-fourths of the study patients were doubly vaccinated against COVID-19 (77; 73.4%), and 20.9% of the patients were partially immunized (received the first dose). A total of 14 (13.3%) patients had a history of COVID-19 infection in the past two weeks prior to current admission, while 33 (31.4%) patients suffered within the past two to four weeks.

**Table 1:** Baseline characteristics of the study population. COVID-19: coronavirus disease 2019

Characteristics		Frequency
Menopausal status	Pre-menopausal	86 (81.9%)
	Post-menopausal	19 (18.1%)
Socio-economic status	Upper	7 (6.7%)
	Upper middle	35 (33.3%)
	Lower middle	31 (29.6%)
	Upper lower	26 (24.7%)
	Lower	6 (5.7%)
Co-morbidities	Hypertension	7 (6.7%)
	Diabetes	12 (11.4%)
	Cardiovascular diseases	3 (2.8%)
	Respiratory diseases	2 (1.9%)
	Steroid-dependent rheumatoid arthritis	1 (0.9%)
Mode of COVID-19 testing	Reverse transcriptase polymerase chain reaction	69 (65.8%)
	Cartridge-based nucleic acid amplification test	24 (22.8%)
	Rapid antigen test	12 (11.4%)
Vaccination status	Not vaccinated	6 (5.7%)
	Partially vaccinated	22 (20.9%)
	Fully vaccinated (two doses)	77 (73.4%)
COVID-19 infection status	History of COVID-19 infection within the past two weeks	14 (13.3%)
	Past two to four weeks	33 (31.4%)

The division of the number of laparoscopic surgeries performed based on the decision to incision interval is depicted in Table 2. Immediate, urgent, and expedited surgeries comprised 11.4%, 22.8%, and 65.8% of total surgeries, respectively. Cases of twisted dermoid cysts, twisted para-ovarian cysts, ruptured ectopic pregnancies, and ruptured tubo-ovarian abscesses were classified as immediate surgeries, with a mean duration from the decision to surgical incision being 0.54 hours (32 minutes). Urgent surgeries comprised hematometra drainage with laparoscopic excision of uterine rudimentary horn, salpingectomies in unruptured and chronic ectopic pregnancies, hysteroscopic polypectomies in patients with heavy menstrual bleeding, and salpingectomy in a patient with pyosalpinx where the mean duration between the decision to surgery was 14.8 hours. Other laparoscopic surgeries, such as laparoscopic hysterectomies and myomectomies for heavy menstrual bleeding, endometriotic cystectomies, hysteroscopic septal resections, and salpingo-oophorectomy in a patient with giant mucinous cystadenoma were categorised into expedited surgeries, with an average time from the decision to surgery being 60.2 hours. On assessing the ease of pre-operative preparation according to the five-point Likert scale, immediate surgeries were rated with a mean score of two suggesting the difficulty faced during the pre-operative period. Problems encountered in getting documented COVID-19 reports and organising the anaesthesia team, nursing staff, and paramedical assistants led to a delay in starting surgery. Urgent and expedited surgeries were relatively easier to prepare (with mean scores of four and five on the Likert scale, respectively).

**Table 2:** Division of the number of laparoscopic surgeries performed based on the decision to incision interval period. COVID-19: coronavirus disease 2019

	Immediate (n = 12; 11.4%)	Urgent (n = 24; 22.8%)	Expedited (n = 69; 65.8%)
Surgeries	Cystectomy in cases of ovarian dermoid with torsion = 6	Laparoscopic excision of rudimentary horn with hematometra = 6	Total laparoscopic hysterectomy due to heavy menstrual bleeding = 27
	Emergency salpingectomy in ruptured ectopic pregnancies = 3	Salpingectomy in unruptured and chronic ectopic pregnancies = 9	Laparoscopic myomectomy due to heavy menstrual bleeding = 23
	Cystectomy of para-ovarian cysts with torsion = 2	Hysteroscopic polypectomy in patients with heavy bleeding due to endometrial polyp = 8	Endometriotic cystectomy in patients with dysmenorrhoea = 16
	Salpingo-oophorectomy in ruptured tubo-ovarian abscess = 1	Salpingectomy in a case of pyosalpinx = 1	Septal resection due to hematometra with severe dysmenorrhoea = 2
			Salpingo-oophorectomy in a complex ovarian cyst (mucinous cystadenoma) = 1
Type of COVID-19 test done	Rapid antigen test	Cartridge-based nucleic acid amplification test	Reverse transcriptase polymerase chain reaction
Mean duration from the decision to surgery (in hours)	0.54 hours	14.8 hours	60.2 hours
Ease of pre-operative preparation (Likert scale)	2	4	5
Average number of persons assisting the primary surgeon	2	2	4
Mean duration of surgery (in minutes)	37.6 minutes	44.2 minutes	92.6 minutes
Mean duration of hospital stay (in hours)	18.4 hours	36.8 hours	96.4 hours
Patient satisfaction (Likert scale)	4	5	5

The average number of persons assisting the chief surgeon was two, two, and three in the immediate, urgent, and expedited group of surgeries, respectively. The mean duration of surgery in the immediate and urgent groups was 37.6 and 44.2 minutes, respectively. The expedited group comprising mostly laparoscopic myomectomies and hysterectomies required a longer surgical duration (average 92.6 minutes). The average duration of hospital stay in the three groups was 18.4 hours, 36.8 hours, and 96.4, hours respectively. Patient satisfaction was also rated according to the Likert scale. The mean ratings of satisfaction by the patients were four, five, and five, respectively, in the three subgroups.

## Discussion

During the first COVID-19 wave, the American Society of Anesthesiologists (ASA) recommended that elective surgeries should be postponed for at least four weeks even in asymptomatic COVID-19-positive patients [6]. In the United States, most elective surgeries were deferred during the first and second COVID-19 waves [10,11]. Only emergency surgeries were allowed. While the infectivity rate of the Omicron variant was comparatively high, intensive care unit (ICU) admissions and mortality due to COVID-19 pneumonia were substantially low [3]. Hence, instead of a blanket recommendation to stop all elective procedures, surgical risk assessment is vital to predicting the value of care of a surgical procedure. Such a comprehensive approach is necessary to implement a modified ‘green pathway’ where vaccinated asymptomatic patients can safely undergo elective surgeries within five to ten days after COVID-19 infection [11,12]. A similar protocol was followed in the present study, and a history of recent COVID-19 infection was not a contraindication for laparoscopic surgery during the Omicron wave. Tummers et al. conducted a literature review to discuss the potential risk of laparoscopy in COVID-19-positive patients undergoing emergency surgeries and to provide guidance for healthcare workers to prevent the transmission risk by useful safety measurements [13]. In the review, they included studies addressing relevant information regarding the SARS-CoV-2 RNA virus, potential hazards of laparoscopy, concerns of viral transmission, and protective measurements during surgery for viral transmission. A total of 26 articles and 15 guidelines were used. The review concluded that laparoscopy using appropriate safety measures remains the first-line management option even during the COVID-19 pandemic.

The Society of American Gastrointestinal and Endoscopic Surgeons (SAGES) and the European Association of Endoscopic Surgery (EAES) recommended safety issues related to laparoscopic surgeries during the ongoing pandemic [14]. All emergency laparoscopic procedures performed during these times should be considered as high risk with mandatory PPE. Although theoretically laparoscopy can cause aerosolisation of blood-borne viruses, no available evidence confirms this with respect to coronavirus [15]. The verdict about the approach should be finalised bearing in mind the established benefits of minimally invasive surgery versus the potential theoretical risks of aerosolisation. However, some studies have shown SARS-CoV-2 virus transmission in surgical smoke during laparoscopic surgery [16]. Therefore, safety precautions should be taken during surgery that consists of operating at a reasonably low intra-abdominal pressure, evacuating the pneumoperitoneum via a filtration system before closure, specimen extraction through the vault after deflating the pneumoperitoneum, using disposable trocars, and using electrosurgical devices at low-power settings. These protocols were strictly followed during all surgeries in our study. The restrictions imposed during the first and second waves taught an important lesson. A restricted number of assistants during surgery, limited paramedical staff, stringent anaesthetic protocols, and a reduced number of operation theatre slots during the past two years have made us receptive to the most vulnerable situations. Resident doctors and supporting paramedical staff are now quite adapted to changed peri-operative management protocols during the pandemic. Although only 60% of the total staff was available for non-COVID-19 hospital care, no emergencies and semi-emergencies were neglected in the Omicron wave.

The milder Omicron wave has been favourable in bringing some amendments to our surgical approach. Operative guidelines were not so stringent. Admission protocols were also liberalised to a great extent. The process of admission to general wards was easier compared to that during the Delta wave. Previously during the Delta wave, an RT-PCR report was mandatory for admission to the wards, but now patients could be admitted and operated on after a RAT as well. Moreover, RT-PCR report from laboratories outside the hospital of admission was also considered valid. Although there was great apprehension in the beginning, the milder nature of the disease and the huge number of vaccinated staff and patients made us revise our existing policies. Pre-operative patient preparation during the Omicron wave was faster, thereby decreasing the decision to incision interval compared to the Delta wave [5]. The peri-operative safety protocols were almost similar, yet the surgical time was decreased without compromising the personal safety measures. More number of vaccinated ground staff, quicker intubation and extubation during surgeries, and lesser duration of the post-operative hospital stay helped significantly increase the total number of surgeries being performed (105 surgeries over three months vs. 60 surgeries over the six months during the Delta wave) [5].

The high level of satisfaction among the patients could be explained by the fact that endoscopic surgeries could be successfully performed in most gynaecological emergencies, thereby leading to a shorter duration of hospital stay and fewer chances of cross infections. Although there were restrictions regarding laparoscopic surgical access in many private centres, these challenges could be dealt with in our multidisciplinary setting. Lesser morbidity post-COVID-19 infection has led to an earlier resumption of duties among resident doctors and paramedical staff. In addition, the post-infection isolation duration was reduced from 14 days to seven days during the Omicron wave. Hence, an ample number of hospital staff could smoothly run the administration during the Omicron wave. Moreover, considering the high proportion of immunisation among both doctors and patients, fear of COVID-19-related stigma had almost dwindled.

The study had a few limitations. It was a non-randomised, single-centre study. Only benign gynaecological surgeries on COVID-19-negative patients were included in the study. No comparison was done between surgeries performed on COVID-19-positive and negative patients as laparoscopic surgeries were restricted to confirmed COVID-19-negative patients only.

## Conclusions

With appropriate screening measures, gynaecological laparoscopic surgeries could be safely performed during the peak of the COVID-19 pandemic. Considering the advantages of laparoscopy, it is the preferred mode of surgery compared to laparotomy even during the COVID-19 pandemic. Through this study, we tried to analyse the lessons learnt from the previous Delta wave and how we could implement those to modify the existing hospital policies during the Omicron wave. It will indeed boost us to get prepared for better patient care during future spikes of the disease.
